# Phthisis in the Boot and Shoe Industry

**Published:** 1915-11-27

**Authors:** 


### PHTHISIS IN THE BOOT AND SHOE INDUSTRY.

### Medical Research Committee. (First Report.)

EVIDENCE of the practical nature of the work done by the Medical Research Committee established under the National Health Insurance Act is now available, and such work makes a strong appeal to the average citizen, for are not the funds employed partly derived from the compulsory contributions of the democracy? Following closely on the first Annual Report of the Medical Research Committee there has now been issued the first Report of a special investigation Committee which is inquiring into the incidence of pulmonary tuberculosis in relation to occupations. This special Committee consists of Drs. Christopher Addison, John Brownlee, E. L. Collis, Leonard Hill, and Benjamin Moore, with the secretary of the parent Committee, Dr. H. M. Fletcher. The particular occupation which has first received their attention is the boot and shoe industry, the chief centres of which are the towns of Leicester and Northampton.

The Report is one which has a general as well as a special interest to the public. The conditions under which the operatives in the different departments usually have to work are fully described, and the determining factors of the high phthisis mortality among boot and shoe operatives are analysed. Among these factors weight is given to the following: The sedentary nature of the employment and the attitude adopted; the presence of infected workers who are by circumstances favourably placed for disseminating bacilli among their neighbours; the lack of sufficient daylight and adequate ventilation; and the presence of infected dust.

The evidence for the statement that boot and shoe operatives suffer a higher prevalence of phthisis than that found for the general population needs very careful handling, but after a minute sifting of complicated statistics, which are reproduced in the tables appended to this Report, the truth of this statement may be accepted.

Turning next to the recommendations of the Committee, we find some account of the chief physiological considerations which are relevant to the points raised. These are in part based upon research work which has been done in the Applied Physiology Department under the Medical Research Committee. Thus the economic as well as hygienic advantage of so-called play intervals is insisted upon. A quarter of an hour interval at 11 A.M. and again at 4 P.M. is suggested, and opportunity should be given for Swedish drill or for games. More and better work is done with than without such intervals. On the subject of ventilation the reporters have attached great weight to the results of recent research, and not only are convinced of the potency for good of fresh air, but demonstrate the economic importance of keeping factories adequately cool.

The important question of sanatorium treatment as applied to the operatives in this industry presents difficulties which all those engaged in tuberculosis will appreciate. The two chief difficulties are: (1) The patient frequently comes under treatment too late; (2) the risk of the patient breaking down again after his return to work and before he has settled down to the pace of the factory. To meet these difficulties a modified form of sanatorium treatment is advocated. Briefly, the scheme is to establish, in connection with the ordinary type of sanatorium, a workplace for the manufacture of boots and shoes. The value of such a scheme, if it be found practicable, requires no demonstration; mentally and physically, work of such a nature is of the greatest help in treatment, and is far superior to the invented, unremunerative physical labour often undertaken in sanatoria. In this scheme patients would work, under medical supervision, for such hours as they are able, and would earn wages in proportion; as health was re-established the working hours and earning capacity would increase, until in many cases factory conditions could be resumed. Employers and workmen are said to have looked favourably on the scheme, but administrative details are not considered as falling within the scope of the report.

Not long ago attention was drawn in THE HOSPITAL to a proposal to establish in Northamptonshire a sanatorium primarily for the use of boot and shoe operatives. It is still possible that the inauguration of such a pioneer movement may take place there. From figures derived from the returns of the medical officer of health for Northampton it is estimated that about 120 cases of phthisis might be expected each year in that district. It is further calculated that the provision of graded work under such a scheme would correspond to the full-time work of about fifty operatives. This seems to forecast that the practical adoption of such a scheme would be distinctly worth while.

